# Interlimb Transfer of Grasp Orientation is Asymmetrical

**DOI:** 10.1100/tsw.2006.291

**Published:** 2006-12-28

**Authors:** Victor Frak, D. Bourbonnais, I. Croteau, H. Cohen

**Affiliations:** ^1^ Département de Kinanthropologie, Université Québec á Montréal, Québec, Canada; ^2^ Institut de Réaptation de Montréal, CRIR Université Montréal, Québec, Canada; ^3^ Memory and Motor Skills, Disorders Research Centre, Clinique Sainte Anne, Québec, Canada

**Keywords:** Prehension, Interlimb transfer, Opposition axis, Hemispheric dominance, Visuomotor transformation

## Abstract

One the most fundamental aspects of the human motor system is the hemispheric asymmetry seen in behavioral specialization. Hemispheric dominance can be inferred by a contralateral hand preference in grasping. Few studies have considered grasp orientation in the context of manual lateralization and none has looked at grasp orientation with natural prehension. Thirty right-handed adults performed precision grasps of a cylinder using the thumb and index fingers, and the opposition axis (OA) was defined as the line connecting these two contact points on the cylinder. Subjects made ten consecutive grasps with one hand (primary hand movements) followed by ten grasps with the other hand (trailing movements). Differences between primary and trailing grasps revealed that each hemisphere is capable of programming the orientation of the OA and that primary movements with the right hand significantly influenced OA orientation of the trailing left hand. These results extend the hemispheric dominance of the left hemisphere to the final positions of fingers during prehension.

## INTRODUCTION

Reaching and grasping an object is a complex motor task involving the proximal and distal joints. One of the most influential theories of prehensile movements in both primates and humans is that proposed by Jeannerod[1]. In this theory, Jeannerod claims that two distinct processing components are involved in prehension movements: one responsible for the transport of the arm to the object (more dependent on proximal segments of the upper limb) and one for the grip of the object (more dependent on distal segments of the upper limb). It has been presumed that interhemispheric communication is required for the coordination of reaching and grasping[2], supporting the notion that each hemisphere has a predominant involvement during reaching to grasp actions. Previous work supports the idea that grasping is not a purely distal phenomenon, as it also involves the proximal segments of the upper limb. For example, Paulignan et al.[3] studied the action of grasping an object located at different positions in the work field. They found that although the grasp orientation was constant for all positions, the shape of the whole arm changed from one position to another. A study by Stelmach et al.[4] also showed that the orientation of the distal part of the movement (finger grip) is not independent from the organization of the proximal part of the movement (reach). In their work, a relatively small change in the orientation of an object, allowing only one possible grasp orientation, resulted in a major reconfiguration of the arm, including wrist pronation and shoulder abduction. Few studies have considered grasp orientation in the context of manual lateralization[5] and none has looked at grasp orientation in free-precision grasp, i.e., with no constraint of finger position on the object. Grasp orientation, defined by the opposition axis (OA), is the final expression of a composite mechanism — reach and grasp — resulting in the controlled precision prehension of an object. In a precision grip formed by the thumb and index fingers, the OA is defined as the line connecting the final position of the fingers on the object. The fingers involved in the grasp represent the effector of the movement and their final position on the object is a main parameter to be controlled for completing a grasp[6]. Location, size (from 3–9 cm), and weight (from 30–300 g) of cylinders do not impact on grasp orientation, which remains stable, with respect to an egocentric reference frame in right-handed individuals[3,7]. In this perspective, the main objective of this study was to examine whether a dominant arm advantage exists in controlling the OA orientation when natural prehension occurs. We used a cylinder where the thumb and index finger could be placed at any position on its surface.

## METHODS

### Subjects

Thirty, healthy, right-handed adults (14 women and 16 men) volunteered to participate in this investigation. Handedness was determined using the ten-item version of the Edinburgh inventory[8]. Only subjects scoring a laterality quotient of 100 were selected. Subjects ranged in age from 19–70 years (mean = 49). Subjects were recruited and tested following ethical considerations in accordance with the Centre for Interdisciplinary Research in Rehabilitation of Montréal procedures. Before the experiment, subjects were given explanations of the methods used. The purpose of the study was revealed to them once the experiment was over.

### Procedure

The subjects were comfortably seated in front of a table. Subjects were asked to reach, grasp, lift, and return to its original position a smooth 300-g resin cylinder (6 cm in diameter, 10 cm high) placed at the center of the table at a distance of 32 cm from the body plane, using a precision grip formed by the thumb and index fingers only. The OA was defined as the line connecting these two contact points on the cylinder and the OA orientation was calculated with a protractor with respect to the frontal plane. Subjects made ten consecutive grasps with one hand (primary hand movements) followed by ten consecutive grasps with the other hand (trailing hand movements). Fifteen subjects performed the first block of grasps with their right hand and the second block with their left hand. This order was reversed for the other 15 subjects. The initial position of the right hand was 13 cm right of the sagittal axis and 13 cm to the left of the axis for left-hand movements.

## RESULTS

Mean OA orientation from an egocentric frame of reference for primary hand movements was 32° (ranging from 24–40°) for the right and 17° (ranging from 6–23°) for the left. The right-hand OA orientation observed here is in agreement with previous experiments with cylinders of different size, weight, and positions[3,7]. Mean OA orientation for trailing hand movements was 26° (ranging from 14–38°) for the right and 32° (ranging from 20–42°) for the left (Fig. 1).

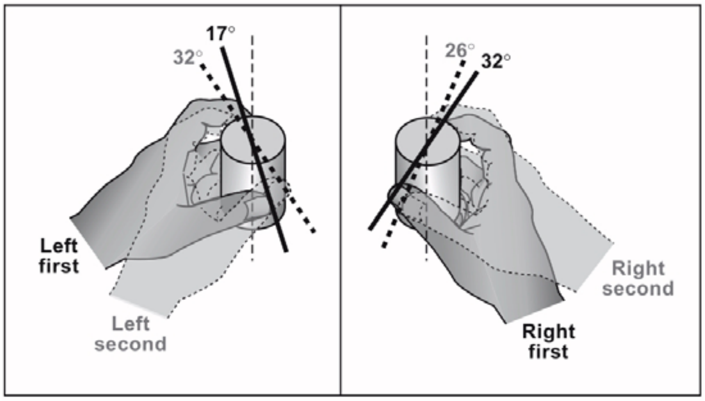

**FIGURE 1.** Mean OA orientation from an egocentric frame of reference for primary hand movements was 32° for the right and 17° for the left. Mean OA orientation for trailing hand movements was 26° for the right and 32° for the left.

A two-factor ANOVA, hand sequence (primary hand, trailing hand) × laterality (right hand, left hand), with repeated measures on both factors and conducted on OA measures, revealed an interaction between hand sequence and laterality ($F(_{1,4}) = 12.06$; $p < 0.0037$) (Fig. 2).

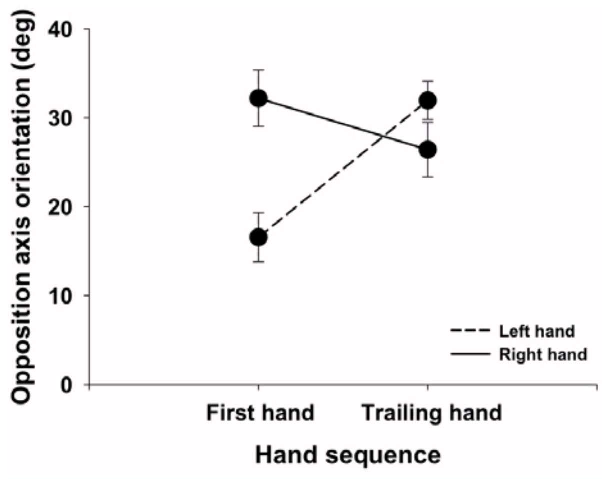

**FIGURE 2.** An interaction between hand sequence and laterality ($F_{(1,4)} = 12.06$; $p < 0.0037$).

Newman-Keuls post hoc multiple comparisons indicated that this interaction was due to significant differences between primary right and left hands ($p < 0.008$). OA orientation was also different between primary and trailing left-hand movements ($p < 0.013$) and between primary left- and trailing right-hand movements ($p < 0.038$). However, there was no difference between primary right- and trailing left-hand ($p = 0.956$) and between primary and trailing right-hand conditions ($p = 0.220$). These results showed that trailing left-hand movements are mirroring right primary hand movements and that the left primary hand has a poor influence on the right trailing hand OA orientation.

No difference in OA orientation was found within each hand trailing condition when comparing the five grasping movements soon after the switch between the hands with the following five trials. Mean OA orientation for trailing hand movements was 27° for the earlier and 26° for the later ($p = 0.11$) grasps for the right hand, and 33° for the earlier and 31° for the later ($p = 0.241$) for the left hand.

## DISCUSSION

The OA orientation was found to differ between primary right and left hands, revealing a specific OA for each hand, suggesting that the left OA orientation is not merely a mirror orientation of the right hand.

It must be noted that the displacement of grasp orientation between the first and second grasp conditions occurs with both subject and object in the same fixed positions throughout the duration of the experiment. This indicates that the final position of the thumb and the index finger is computed from an egocentric reference frame and not from an invariant visual landmark on the object. These observations extend to the left hand previous results seen with the right hand[3]. Thus, we understand that both hemispheres are capable of contralateral motor programming. However, there is a clear advantage of the left hemisphere over the right; the left hemisphere can alter the motor programming of the ipsilateral upper limb to reach a similar orientation, but with different points of contact.

The visuomotor transformation to grasp requires coding of the object's intrinsic properties (size and shape) and the transformation of these properties into a pattern of distal movements[9]. A nondominant hemisphere/limb system has been proposed for controlling static limb position to specify the final position of a reaching movement[10]. These authors show, for some target positions, that the nondominant hand reached the target with smaller final position errors than the dominant hand. Sainburg’s model[11] is supported on experiments that have calculated intersegmental dynamics on reaching to pointing, at a single circle in a plane. However, grasp orientation in a precision grip is defined by two contact points in a three-dimensional space. As the prehension of a cylinder necessitates a grasping orientation computed from an egocentric referential frame and not from a point on the object, our results do not contradict the notion related to the nondominant hemisphere advantage in final position seen in experimentally induced final position.

In some cases, the preferred hand does not constantly produce the best performance. For example, Kimura and Vanderwolf [12] found that during isolated flexion of a single sequence of digits, the task was performed better with the nondominant hand in right handers. Also, Carey et al.[13] showed that subjects are more accurate at an index finger tracking task with their nondominant hand compared to the dominant hand. In the present research, since no difference in grasping performance was observed between the right and left hands, without once letting the cylinder slip or drop over the duration of the experiment, this suggests that it is hand preference and not efficiency that impacts on the change in OA orientation during cylinder grasp.

The first grasping movements soon after the switch between the hands were not influenced more than those in the later trials, indicating that the change induced in the left-grasp orientation has been integrated from the beginning in the motor programming and during the experiment. The fact that the OA of the primary left hand is different from the OA of the primary right hand indicates that this influence is transitory. To date, there is ample evidence showing bilateral CNS involvement during unimanual motor acts (for an extensive review, see Carson[14]). For the duration of the experiment, the displacement of the left OA orientation reflects a mirror movement produced in-phase, i.e., there is a facilitation of the same muscle group. These behavioral observations would support an asymmetrical transfer via a transcallosal facilitatory connection between the upper limb motor areas in the two hemispheres[15].
